# Supplementary material for: LIS1 determines cleavage plane positioning by regulating actomyosin-mediated cell membrane contractility
Source: eLife. 2020 Mar 11;9:e51512. doi: 10.7554/eLife.51512 (PMC7112955; doi:10.7554/eLife.51512)
Supplement: Figure 1—source data 1. [file elife-51512-fig1-data1.docx]

**Figure 1 – Source Data 1.** Quantification of apical NPCs (RGs)

|  | **Symmetric cleavage** | **Asymmetric cleavage** |
| --- | --- | --- |
| **WT*-MADM*** (n=18) | 14 (77.8%) | 4 (22.2%) |
| ***Pafah1b1-MADM: Pafah1b1 +/+*** (n=20) | 15 (75%) | 5 (25%) |
| ***Pafah1b1-MADM: Pafah1b1 ko/+*** (n=24) | 3 (12.5%) | 21 (87.5%) |
| ***Pafah1b1-MADM: Pafah1b1 ko/ko*** (n=20) | 0 (0%) | 12 (60%)  8, Mitotic arrest (40%) |

n: total number of apical NPCs observed in the immunohistochemistry experiments

to detect Anillin distribution
