# Supplementary material for: LIS1 determines cleavage plane positioning by regulating actomyosin-mediated cell membrane contractility
Source: eLife. 2020 Mar 11;9:e51512. doi: 10.7554/eLife.51512 (PMC7112955; doi:10.7554/eLife.51512)
Supplement: Figure 2—source data 1. [file elife-51512-fig2-data1.docx]

**Figure 2 – Source Data 1.** Quantification of apical NPCs (RGs)

|  | **Vertical cleavage plane**  (vertical mitotic spindle) | **Oblique cleavage plane**  (oblique mitotic spindle) |
| --- | --- | --- |
| ***Pafah1b1^hc/+^*** (n=25) | 21 (84%) | 4 (16%) |
| ***GFAP-Cre; Pafah1b1^hc/+^*** (n=25) | 11 (44%) | 14 (56%) |

n: total number of apical NPCs observed in the immunohistochemistry experiments
