# Supplementary material for: LIS1 determines cleavage plane positioning by regulating actomyosin-mediated cell membrane contractility
Source: eLife. 2020 Mar 11;9:e51512. doi: 10.7554/eLife.51512 (PMC7112955; doi:10.7554/eLife.51512)
Supplement: Figure 3—source data 1. [file elife-51512-fig3-data1.docx]

**Figure 3 – Source Data 1.** Quantification of aPKCζ inheritance in apical NPCs (RGs)

|  | ***Pafah1b1^hc/+^*** (n=27) | ***Pafah1b1^hc/ko^*** (n=32) |
| --- | --- | --- |
| **Equal inheritance** | 60.9% | 31.2% |
| **Unequal inheritance** | 39.1% | 68.8% |

n: total number of apical NPCs observed in the immunohistochemistry experiments
