# Supplementary material for: LIS1 determines cleavage plane positioning by regulating actomyosin-mediated cell membrane contractility
Source: eLife. 2020 Mar 11;9:e51512. doi: 10.7554/eLife.51512 (PMC7112955; doi:10.7554/eLife.51512)
Supplement: Figure 4—source data 1. [file elife-51512-fig4-data1.docx]

**Figure 4 – Source Data 1.** Quantification of MEFs

| **A. Completion of cytokinesis** | ***Pafah1b1^+/+^***  (N=4)  85.4 ± 6.1% | ***Pafah1b1^hc/ko^***  (N=6)  18.7 ± 8.0% | **Student’s *t*-test**  ***p*-value** (********p*=0.0004) |
| --- | --- | --- | --- |
| **B. Binucleation** | ***Pafah1b1^+/+^***  (N=4)  5.1 ± 3.1% | ***Pafah1b1^hc/ko^***  (N=6)  40.1 ± 1.2% | (********p*<0.00001) |
| **D. Completion of cytokinesis** | ***CAGG-CreERT2; Pafah1b1^+/+^***  (N=3)  69.4 ± 4.3% | ***CAGG-CreERT2; Pafah1b1^hc/hc^***  (N=4)  36.6 ± 5.3% | (*******p*=0.0061) |
| **E. Binucleation** | ***CAGG-CreERT2; Pafah1b1^+/+^***  (N=3)  15.7 ± 6.1% | ***CAGG-CreERT2; Pafah1b1^hc/hc^***  (N=3)  25.8 ± 6.4% | (ns, *p*=0.2940) |

N: total number of independent experimental sets performed for the time lapse live-cell imaging experiments of MEFs, total mitotic events/cell numbers were monitored;

*Pafah1b1^+/+^* (n=96), *Pafah1b1^hc/ko^* (n=36), *CAGG-CreERT2; Pafah1b1^+/+^* (n=20), *CAGG-CreERT2; Pafah1b1^hc/hc^* (n=27)
