# Supplementary material for: LIS1 determines cleavage plane positioning by regulating actomyosin-mediated cell membrane contractility
Source: eLife. 2020 Mar 11;9:e51512. doi: 10.7554/eLife.51512 (PMC7112955; doi:10.7554/eLife.51512)
Supplement: Figure 5—source data 1. [file elife-51512-fig5-data1.docx]

**Figure 5 – Source Data 1.** Quantification of MEFs

| **C. Hyper-contractility**  **phenotypes** | ***Pafah1b1^+/+^***  (N=4)  3.6 ± 3.6% | ***Pafah1b1^hc/ko^***  (N=5)  56.2 ± 8.4% | **Student’s *t*-test**  ***p*-value** (*******p*=0.0011) |
| --- | --- | --- | --- |
| **E. Hyper-contractility**  **phenotypes** | ***CAGG-CreERT2; Pafah1b1^+/+^***  (N=3)  13.5 ± 2.4% | ***CAGG-CreERT2; Pafah1b1^hc/hc^*** (N=4)  40.5 ± 8.4% | (*******p*=0.0446) |

N: total number of independent experimental sets performed for the time lapse live-cell imaging experiments of MEFs, total mitotic events were monitored; *Pafah1b1^+/+^* (96), *Pafah1b1^hc/ko^* (36), *CAGG-CreERT2; Pafah1b1^+/+^* (20), *CAGG-CreERT2; Pafah1b1^hc/hc^* (27)
