# Supplementary material for: LIS1 determines cleavage plane positioning by regulating actomyosin-mediated cell membrane contractility
Source: eLife. 2020 Mar 11;9:e51512. doi: 10.7554/eLife.51512 (PMC7112955; doi:10.7554/eLife.51512)
Supplement: Figure 6—source data 1. [file elife-51512-fig6-data1.docx]

**Figure 6 – Source Data 1.** Quantification of MEFs

| **E. Anillin Distribution** | ***Pafah1b1^hc/+^*** (n=65) | ***Pafah1b1^hc/ko^*** (n=48) |
| --- | --- | --- |
| **normal** | 72.3% | 27.1% |
| **asymmetric** | 10.8% | 14.6% |
| **dispersed** | 15.4% | 41.7% |
| **central MT** | 1.5% | 16.6% |

n: total number of MEFs observed in the immunocytochemistry experiments
