# Supplementary material for: LIS1 determines cleavage plane positioning by regulating actomyosin-mediated cell membrane contractility
Source: eLife. 2020 Mar 11;9:e51512. doi: 10.7554/eLife.51512 (PMC7112955; doi:10.7554/eLife.51512)
Supplement: Figure 8—source data 1. [file elife-51512-fig8-data1.docx]

**Figure 8 – Source Data 1.** Quantification of MEFs

| **E. Actomyosin Distribution** | ***Pafah1b1^hc/+^*** (N=6) | ***Pafah1b1^hc/ko^*** (N=4) |
| --- | --- | --- |
| **normal** | 73% | 30% |
| **dispersed** | 27% | 70% |

N: total number of MEF immunocytochemistry experimental sets used for examining Actomysoin distribution. Total fifty cells were counted from all experimentsz
