# Supplementary material for: LIS1 determines cleavage plane positioning by regulating actomyosin-mediated cell membrane contractility
Source: eLife. 2020 Mar 11;9:e51512. doi: 10.7554/eLife.51512 (PMC7112955; doi:10.7554/eLife.51512)
Supplement: Figure 10—source data 1. [file elife-51512-fig10-data1.docx]

**Figure 10 – Source Data 1.** Quantification of MEFs

| **C. SEPT6-GFP**  **Cytokinetic Failure** | ***CAGG-CreERT2; Pafah1b1^+/+^*** (n=7)  1 (14.3%) | ***CAGG-CreERT2; Pafah1b1^hc/hc^*** (n=6)  4 (66.7%) |
| --- | --- | --- |

n: total number of MEFs observed in live-cell imaging of SEPT6-GFP during cytokinesis
