# Supplementary material for: LIS1 determines cleavage plane positioning by regulating actomyosin-mediated cell membrane contractility
Source: eLife. 2020 Mar 11;9:e51512. doi: 10.7554/eLife.51512 (PMC7112955; doi:10.7554/eLife.51512)
Supplement: Figure 11—source data 1. [file elife-51512-fig11-data1.docx]

**Figure 11 – source data 1.** Quantification of MEFs

| **E. Anillin Distribution** | ***Pafah1b1^+/+^***  **+ GFP**  (n=23) | ***Pafah1b1^+/+^***  **+ CA-RhoA**  **-GFP**  (n=20) | ***Pafah1b1^hc/ko^***  **+ GFP**  (n=24) | ***Pafah1b1^hc/ko^***  **+ DN-RhoA**  **-GFP**  (n=24) |
| --- | --- | --- | --- | --- |
| **normal** | 78.3% | 22% | 25% | 70.8% |
| **asymmetric** | 8.7% | 22% | 33.3% | 12.5% |
| **dispersed** | 13.0% | 56% | 33.3% | 16.7% |
| **central MT** | 0% | 0% | 8.3% | 0% |

n: total number of MEFs observed in immunocytochemistry to detect Anillin distribution
